# Open question: Can Chlamydia pneumoniae cause persistent infections, and how are these associated with chronic diseases?

**DOI:** 10.1099/jmm.0.002200

**Published:** 2026-09-03

**Authors:** David L. Hahn

**Affiliations:** 1 Intracell Research Group, Wake Forest, NC, USA

**Keywords:** *Chlamydia pneumoniae*, chronic infection, healthy blood donors

I would like to comment on some aspects of an excellent and informative review that I recently came across, published in the *Journal of Medical Microbiology* (Tagini F, Puolakkainen M, Greub G, On Behalf of the Escmid Study Group for Mycoplasma and Chlamydia Infections (ESGMAC). From coughs to complications: the story of *Chlamydia pneumoniae*. *J Med Microbiol* 2025;74) [1]. The authors note the regrettable decline in research on *C. pneumoniae* and end their review with a list of ‘open questions’ that are ripe for research. I would like to comment on one of these questions and provide some additional supporting data.

The authors’ review includes the biological propensity for *C. pneumoniae* to produce persistent infections capable of eliciting chronic inflammation in the infected host; they also review the fairly large body of cross-sectional epidemiological associations of various *C. pneumoniae* biomarkers with several chronic inflammatory conditions of currently unknown aetiology: asthma, Alzheimer’s dementia and atherosclerosis. Beyond cross-sectional associations, there is a paucity of prospective human clinical, microbiological and therapeutic studies into any of these conditions; hence the question: can *C. pneumoniae* cause persistent infections, and how are these associated with chronic diseases? It is this question I would like to address in this Letter.

First, can *C. pneumoniae* cause persistent infections?

Seroepidemiological studies find that infections begin in childhood and the majority of adults worldwide remain seropositive, suggesting that reinfections and/or chronic persistent infections are widespread [2]. Histopathological tissues from children and adults confirm that deep tissue infection is present in a large proportion of individuals studied. In children, the first study of adenoid tissue removed at surgery was positive by immunohistochemical staining for *C. pneumoniae* in 68 of 69 children, and others subsequently confirmed positivity albeit at lower proportions [3]. In adults, a study by Wu *et al*. [4] examined lung tissue resections for cancer, in which 100% of samples obtained from non-diseased areas tested positive by immunohistochemical staining for *C. pneumoniae*. Staining was seen exclusively in monocytes within the lung, and samples from individuals with comorbid chronic obstructive pulmonary disease (COPD) had the greatest infection burdens. (This latter finding may be relevant considering that azithromycin is now a guideline-recommended treatment to reduce exacerbations in COPD, although its mechanism of action remains controversial [5].) Comparable studies showing positive results in immune cells in the brain and vascular system also exist [6, 7]. Strikingly, Wu *et al*. [4] also reported that 41% of their samples from young adult and middle-aged trauma victims also stained positive for *C. pneumoniae* within lung monocytes, albeit at a lower infection burden than lung cancer samples. Direct organism identification in human donor blood provides further compelling evidence for *C. pneumoniae* persistence (Table 1). Of the 12 studies summarized in Table 1, the range of positive *C. pneumoniae* detection ranged from 3.6% to 63.3% (weighted average, 23.3%). Taken together, the high prevalence of detectable *C. pneumoniae* within multiple organ systems of both healthy and diseased hosts plus frequent detection in peripheral blood mononuclear cells of apparently healthy blood donors is clear proof that the answer to the question is: yes, *C. pneumoniae* can definitely cause persistent infections, and so frequently that one wonders how often this *intracellular* organism causes disease versus serving as a commensal! These data also support the hypothesis that tissue reservoirs of infection within mobile mononuclear cells intermittently seed the peripheral circulation. The blood donor data raise several questions, among which are:

- Why does blood PCR positivity persist in apparently healthy adults, most of whom exhibit protective antibodies? Like other Chlamydiae, *C. pneumoniae* is notorious for producing persistent and chronic intracellular infections [8]. *C. pneumoniae* chronic infection resides in a metabolically inactive intracellular form that is relatively protected from the humoral (extracellular) immune response [8]. Factors governing why and how often intermittent trafficking of infected monocytes occurs from tissue reservoirs (such as the lung, vascular system and brain) to the general circulation are unknown, but could be regulated by cellular immunity. In addition to humoral immunity, therefore, cell-mediated immunity (CMI) testing should be included to study whether CMI plays a role in trafficking.
- Why is the detection range so broad? Variations in population infection burden, trafficking and PCR sensitivities are likely explanations. As much as 100-fold differences in sensitivities of various PCR tests have been documented [9]. For example, a single-step PCR yielded no positive results, but a nested PCR showed frequent *C. pneumoniae* positivity in a childhood respiratory study (S. Johnston, personal communication) [10]. Furthermore, not all lab-based PCR tests have been adequately validated [11].
- Can peripheral blood be used as a surrogate for infection elsewhere in the body? To my knowledge, other than for chronic bronchitis [12], this question has not been studied. A research consortium has outlined a proposal and a call for collaboration to establish a consensus protocol to explore the brain pathobiome in patients with mild cognitive impairment and Alzheimer’s disease; the utility of easily obtained biological samples, such as peripheral blood, as markers for brain infection is among their aims [13]. Chlamydia researchers are actively involved in this initiative.

**Table 1.** *C. pneumoniae* in peripheral blood from healthy donors

Reference and country	Method	Results positive	Additional comments
Boman [15] Sweden	MOMP touchdown nPCR of PBMCs	24/52 (46.2%)	24/24 PCR+ were IgG MIF+ and 18/24 (75%) were IgA MIF+
Bodetti [16] Australia	OmpA PCR on PBMCs; IF staining of PBMCs	10/60 (16.7%)	Ten positive by both techniques
Kaul [17] USA	Omp1/hsp60 touchdown nPCR of PBMCs	5/19 (26.3%)	Two of five PCR positives were IgG seronegative
Haranaga [18] USA	16S rRNA touchdown PCR of PBMCs	21/237 (8.9%)	Seasonal variation noted
al-Amro [19] Saudi Arabia	Unstated primer PCR of PBMCs	1/28 (3.6%)	Samples were from a military hospital and 26 of 28 were male
Rassu [20] Italy	OmpA touchdown nPCR of PBMCs	78/169 (46.2%)	On retesting 8 months later, 26/38 (68%) initial PCR+ donors turned negative and 13/28 (46%) initial PCR− donors turned positive; 15/44 (34.1%) PCR+ donors who had serological results were seronegative; both monocytes and lymphocytes were infected
Fukano [21] Japan	53 kDa nPCR of PBMCs	18/77 (23.4%)	Four other PCRs had lesser detection rates ranging from 0–9.1%
Yamaguchi [22] Japan	16S rRNA RT-PCR on PBMCs	13/70 (18.6%)	No differences in IgG (ELISA) for PCR+ vs PCR− samples
Ikejima [23 USA	16S rRNA real-time PCR on whole blood	19/30 (63.3%)	One of 12 PCR-positive RBC units remained PCR+ after leukoreduction
Cirino [24] USA	Culture of buffy coat	114/459 (24.8%)	IF staining of fresh blood smears was positive in 113/459 (24.6%); inclusions seen in neutrophils, monocytes and eosinophil/basophil cells.
Webley [25] USA	Culture of buffy coat	35/115 (30.4%)	IF staining of fresh blood smears was positive in 31/115 (27.0%) before leukoreduction and in 2/115 (1.7%) after leukoreduction. Culture was positive in 1/115 (0.9%) after leukoreduction.
Karimi [26] Iran	16S rRNA RT-PCR of PBMCs	14/196 (7.1%)	
Total		352/1512 (23.3%)	

Results of a PubMed search spanning 1989 to 24 August 2025 using the terms (Chlamydophila pneumoniae OR Chlamydia pneumoniae) AND (Blood Donors OR Transfusion).

hsp60, heat shock protein 60; MIF, microimmunofluorescence serological test; MOMP, major outer membrane protein; nPCR, nested polymerase chain reaction; Omp1, outer membrane protein 1; OmpA, outer membrane protein A; PBMC, peripheral blood mononuclear cells; RT PCR, reverse transcriptase PCR; 16S rRNA, 16S subunit of bacterial ribosomal RNA.

- Does the high prevalence of *C. pneumoniae* positivity among ostensibly healthy blood donors preclude finding associations? As mentioned previously, persistent intracellular *C. pneumoniae* infections can be detected within tissues of both diseased and non-diseased human hosts. Solid organ-based persistent *C. pneumoniae* organisms residing within mobile host immune cells likely traffic into the peripheral circulation. Whether this shedding is proportionate to tissue burden, is completely random and unpredictable or is triggered by measurable host characteristics are unknown questions ripe for research. On the one hand, high background population prevalence of any biomarker may decrease power to find significant differences. On the other hand, it may be that current positivity in apparently healthy individuals is actually a risk factor for later development of chronic disease. In the latter scenario, the high prevalence of positivity may increase power to obtain conclusive evidence for or against causation in prospective cohort studies.

Second, how is *C. pneumoniae* persistent infection associated with chronic diseases?

The purpose of this Letter is to reinforce the message of Tagini *et al*. [1] that it is important to implement research into *C. pneumoniae* as a potential contributor to morbidity of several chronic conditions. From my epidemiological and clinical perspectives, I foresee two research approaches to investigate whether persistent intracellular *C. pneumoniae* infections cause human disease: (i) prospective clinical and microbiological epidemiological cohort studies [7] and (ii) randomized treatment trials that include infection biomarkers to test whether biomarker status predicts a favourable outcome. Both these research approaches require development of a consensus protocol to evaluate the utility of peripheral biomarkers as indicators of deep tissue chronic infection. A different, entirely speculative, ‘biomarker’ approach involves organ positron emission tomography (PET), assuming that infection-specific markers detectable by PET scanning can be developed [14].

I propose development of a consensus protocol for the indirect microbiological diagnosis of chronic *C. pneumoniae* infection comparable to what has been proposed for the study of Alzheimer’s dementia [13]. The Alzheimer’s protocol proposes a one-disease-to-many-pathogens model; a *C. pneumoniae* protocol proposes a one-pathogen-to-many-diseases model. Both approaches utilize similar techniques and tactics, among which are, initially, tissue pathological studies such as those mentioned above, and also therapeutic trials to determine the value of peripheral infection biomarkers as predictors of treatment outcome. An example is iTREAT, an ongoing trial of azithromycin in asthma that will study peripheral blood *C. pneumoniae* PCR and serology as potential predictors of treatment success (https://itreatasthma.org/). Positive results would have a major beneficial impact on a chronic inflammatory disease of unknown aetiology that was not thought to be related to infection. Other *C. pneumoniae*-associated diseases deserve equal attention. Prospects for treatment and even prevention by early diagnosis seem profound.

## Abbreviations

CMI: cell-mediated immunity
COPD: chronic obstructive pulmonary disease
PET: positron emission tomography
